# Ferritin in Kidney and Vascular Related Diseases: Novel Roles for an Old Player

**DOI:** 10.3390/ph12020096

**Published:** 2019-06-21

**Authors:** József Balla, György Balla, Abolfazl Zarjou

**Affiliations:** 1 HAS-UD Vascular Biology and Myocardial Pathophysiology Research Group, Hungarian Academy of Sciences, H-4032 Debrecen, Hungary; balla@belklinika.com (J.B.); balla@med.unideb.hu (G.B.); 2 Division of Nephrology, Department of Medicine, Faculty of Medicine, University of Debrecen, H-4032 Debrecen, Hungary; 3 Department of Pediatrics, Faculty of Medicine, University of Debrecen, H-4032 Debrecen, Hungary; 4 Department of Medicine, University of Alabama at Birmingham, Birmingham, AL 35294, USA; 5 Nephrology Research and Training Center, University of Alabama at Birmingham, Birmingham, AL 35294, USA

**Keywords:** iron, ferritin, acute kidney injury, chronic kidney disease, vascular calcification

## Abstract

Iron is at the forefront of a number of pivotal biological processes due to its ability to readily accept and donate electrons. However, this property may also catalyze the generation of free radicals with ensuing cellular and tissue toxicity. Accordingly, throughout evolution numerous pathways and proteins have evolved to minimize the potential hazardous effects of iron cations and yet allow for readily available iron cations in a wide variety of fundamental metabolic processes. One of the extensively studied proteins in the context of systemic and cellular iron metabolisms is ferritin. While clinicians utilize serum ferritin to monitor body iron stores and inflammation, it is important to note that the vast majority of ferritin is located intracellularly. Intracellular ferritin is made of two different subunits (heavy and light chain) and plays an imperative role as a safe iron depot. In the past couple of decades our understanding of ferritin biology has remarkably improved. Additionally, a significant body of evidence has emerged describing the significance of the kidney in iron trafficking and homeostasis. Here, we briefly discuss some of the most important findings that relate to the role of iron and ferritin heavy chain in the context of kidney-related diseases and, in particular, vascular calcification, which is a frequent complication of chronic kidney disease.

## 1. The Superfamily of Ferritins

The ability of iron cations (the second most abundant element on earth) to change its valence serves as a unique and central capacity to contribute in multiple biological pathways, yet underscores its potential hazardous effects mainly by participating in Fenton’s reaction [1,2].

O_2_^●−^ + Fe^3+^←→O_2_ + Fe^2+^
Fe^2+^ + H_2_O_2_→Fe^3+^ + HO^●^ + OH^−^ (Fenton’s reaction)
O_2_^●−^ + H_2_O_2_←→O_2_ + HO^●^ + OH^−^ (Haber–Weiss reaction)

The discovery of ferritin, a protein isolated from horse spleen, was a major step in our understanding of iron metabolism [3]. This discovery was followed by numerous studies that examined this multifaceted protein in various aspects of physiological and pathological conditions [4]. The highly conserved structure of ferritin and its universal expression among species further highlights the importance of this protein at the crossroads of multiple biological pathways that are mainly dictated by iron trafficking and homeostasis [4,5,6]. Ferritin is a globular protein made up of 24 subunits with a spherical hollow shell that allows for the safe storage of up to 4500 atoms of Fe^3+^. These subunits are composed of heavy (FtH) and light (FtL) chains and their proportional contribution to the hollow spherical shell depends on the tissue and developmental stage of the organism [5,7]. For instance, while brain and heart ferritin is primarily composed of FtH chains, the liver and spleen mainly possess FtL [7]. One intriguing aspect of ferritin biology relates to serum ferritin that is predominantly composed of FtL chain, as evidenced by immunological cross reactivity with anti-ferritin L antibodies [8,9,10]. Serum ferritin is also relatively iron poor and its source was not completely understood until it was revealed by Cohen and colleagues that macrophages are the primary source of serum ferritin [11]. It must be noted that several investigators have reported that other cells, particularly hepatocytes, are also capable of secreting ferritin [12,13,14]. The study by Cohen et al. elegantly confirmed the light chain predominance of serum ferritin and its relatively low iron cations content, and provided evidence to support its secretion through the non-classical lysosomal secretory pathway [11]. More recently, another study reported that secretion of ferritin is mediated through two distinct non-classical pathways [15]. This study also examined the release of iron-rich ferritin and demonstrated that such secretion occurs via the multivesicular body-exosome pathway [15]. The release of iron-rich ferritin is particularly interesting as it may serve as an iron cargo predominantly in a paracrine fashion. The receptors, precise mode of uptake, and relevance of ferritin uptake by cells among species continues to be debated in the literature and future studies are needed to unequivocally describe the nature and significance of these processes.

Serum ferritin has long been used by clinicians to assess body iron stores in humans. However, it is also recognized that levels of serum ferritin rise in response to a number of clinical conditions particularly during inflammatory states and injury models, such as AKI [16,17,18]. Such elevation in response to inflammatory stimuli and the relatively iron-poor status of serum ferritin resulted in investigations to examine other potential roles in health and disease that were reviewed by Wang and colleagues [19]. More recently, an interesting pilot study examined the feasibility of measuring urinary ferritin (FtL) as a non-invasive diagnostic approach to assess neonates and young children for iron deficiency or iron overload [20]. This study was based on previous reports that confirmed the presence of urinary ferritin in healthy individuals [21,22] and, despite some limitations, found a correlation with paired serum ferritin. In contrast to FtL, the FtH subunit has crucial ferroxidase activity that converts Fe^2+^ to Fe^3+^, facilitating its safe storage in the form of mineral ferrihydrite within the inner wall of the sphere [23,24]. Such functional properties have led to a significant amount of investigations into this field, leading to the discovery of seminal findings into the role of this superfamily of proteins in physiological processes and injury models [4,6,25,26]. 

The first description of a function for ferritin other than mere iron cation storage was reported by Balla and colleagues [27]. This seminal manuscript described an anti-oxidant role for ferritin in endothelial cells. In this study authors validated the cytotoxic properties of heme, but surprisingly found that briefly pulsing cells with heme remarkably increased their resistance against oxidant-mediated injury. While administration of apoferritin mimicked these beneficiary effects, a site-directed mutant form of FtH with subsequent loss of ferroxidase activity failed to recapitulate such protection against oxidative damage [27]. These findings generated significant interest in this field and were followed by additional pivotal findings. For instance, while ferritin was traditionally regarded as a cytosolic protein, others demonstrated its localization in other subcellular compartments, such as mitochondria [28,29] and the nucleus [30,31,32]. This review is intended to briefly discuss some of the aspects of ferritin in the context of kidney-related diseases and one of its major cardiovascular complications, namely vascular calcification.

## 2. Role of Iron and Ferritin in Acute Kidney Injury

Acute kidney injury (AKI) is a common clinical syndrome characterized by a sudden decrement in kidney function with ensuing derangements in multiple essential physiological parameters, such as dysregulated volume and electrolyte homeostasis [33,34]. It is well documented that AKI increases morbidity and mortality, particularly among critically ill patients [33,35,36]. Moreover, AKI incidence is on the rise and patients who survive AKI have an increased risk for the development of chronic kidney disease (CKD) [37,38,39]. These detrimental clinical consequences have led to vigorous investigations to better understand the individual and overlapping pathways that are involved in the pathogenesis of AKI. Iron metabolism and trafficking has emerged as one such pathway. The kidney was not considered a major organ involved in the regulation of iron homeostasis, mainly because of the lack of evidence of its involvement in hereditary hemochromatosis and the assumption that transferrin bound iron cations are not filtered by the glomerulus. However, along with other recent breakthroughs in the field of iron metabolism, our understanding of the delicate and well-orchestrated iron regulatory machinery in the kidney has evolved thanks to a number of pivotal observations. For example, the urinary proteomic analysis of Fanconi syndrome, which is characterized by overall dysfunctional proximal tubules and poor reabsorption capacity, demonstrates significantly elevated levels of transferrin in the urine samples of subjects, confirming its filtration [40,41]. Transferrin can be reabsorbed in the tubules via transferrin receptor-1 or cubilin-mediated endocytosis [42,43]. The kidneys also express high levels of divalent metal transporter-1, which was first described by Gunshin et al. [44], and their importance in renal iron handling under physiological and pathological conditions was subsequently confirmed by other investigations [45,46,47,48,49]. Some other iron regulatory proteins that are expressed in kidneys include neutrophil gelatinase-associated lipocalin [50], ferroportin [51,52], as well as hepcidin [53]. Additionally, among other tissues, mouse kidney expresses the highest levels of iron regulatory protein-1 with important functions in renal iron cations handling [54]. It must also be noted that the proximal tubules, workhouse of nephrons, possess a significant amount of mitochondria that contain notable amounts of heme, which encloses iron cations in its porphyrin ring. Based on the aforementioned evidence, it is apparent that the kidneys, particularly proximal tubules, are heavily involved in iron trafficking under both physiological and pathological conditions, and are accordingly more susceptible to injury [55]. In fact, several lines of evidence suggest a role for iron cations-mediated kidney injury in both humans and animal models of kidney disease [56,57,58,59]. The participation of iron cations in Fenton’s reaction and their ability to aggravate oxidative stress led to the hypothesis that their removal would prove beneficial in kidney injury. In support of this premise, multiple investigations demonstrated that the removal of iron cations via deferoxamine (DFO), a potent iron cations chelator, was protective against AKI. These studies utilized DFO in the setting of glycerol-induced rhabdomyolysis [60], hemoglobin- and myoglobin-induced AKI [61], gentamicin nephrotoxicity [62], and ischemia-reperfusion (I/R) [63]. Notably, these salutary effects were recapitulated by the administration of apotransferrin in a model of I/R-mediated AKI [64]. The evidence to suggest iron as a main culprit and mediator of AKI has also been demonstrated in human studies. Work by Leaf and colleagues focused on the association between catalytic iron cations, non-transferrin bound iron cations, and the course of AKI in patients undergoing cardiac surgery with cardiopulmonary bypass [65]. They reported that primary outcomes, defined as in-hospital death or need for renal replacement therapy, AKI, and other adverse events post-operatively, were directly associated with higher plasma catalytic iron cation levels on post-operative day one [65]. In another study, the same group examined such association in 121 critically ill patients admitted to medical or surgical intensive care units [66]. Similarly, higher plasma catalytic iron cation levels were associated with a greater risk of death/need for renal replacement therapy, AKI, and hospital mortality. Importantly, the authors stated that these associations are independent of age, estimated glomerular filtration rate, and number of packed red blood cell transfusions [66]. Others also evaluated the role of plasma catalytic iron cations in relation to contrast-induced nephropathy. Their results confirmed higher levels of catalytic iron cations were associated with a higher risk of contrast nephropathy, as well as higher rates of mortality [67]. Other studies have suggested a role for catalytic iron cations in the context of glomerulopathies and proteinuria. For instance, in a rat model of nephrotic syndrome, a significant increase in the levels of glomerular catalytic iron cations was observed [68]. Importantly, chelation of iron cations with DFO was associated with complete protection against proteinuria [68]. Another clinical study validated these findings, where deferiprone, an oral iron cation chelator, was used in diabetic and non-diabetic glomerular disease [69]. While this study was rather small and non-randomized, iron cation chelation demonstrated a marked reduction of proteinuria in both groups of patients with and without diabetic glomerular nephropathy.

The above-mentioned evidence led to further investigations to examine how the proximal tubular expression of ferritin may affect the course of AKI. The importance and non-redundant function of FtH and its ferroxidase activity was reported by Ferreira and colleagues [70]. They demonstrated that global deletion of FtH in mice leads to embryonic lethality between 3.5 and 9.5 days of development [70]. Therefore, to understand the role of proximal tubule FtH expression during AKI, we generated transgenic mice with conditional deletion of FtH in proximal tubules. This was achieved by crossing FtH^lox/lox^ mice that were generated and characterized by Darshan and colleagues [71] with phosphoenolpyruvate carboxykinase (PEPCK)-Cre mice [72]. Targeted deletion of FtH in proximal tubules led to heightened rhabdomyolysis-induced AKI, as evidenced by the higher rate of mortality, serum creatinine, and the higher expression of cleaved caspase-3, a marker of apoptosis [18]. The protective nature of FtH expression in proximal tubules was further supported by a histological analysis that revealed a greater number of tubular casts, necrotic tubules, and more prominent loss of proximal tubule brush border in the absence of FtH expression. Notably, rhabdomyolysis is primarily a heme-mediated injury model, given the significant amount of heme present in myoglobin. Therefore, to investigate whether FtH may play a protective role in other models of AKI that are not predominantly heme driven, cisplatin nephrotoxicity was used. These results indicated that, irrespective of the model of injury, deletion of FtH led to worse renal function, as evidenced by serum creatinine. Furthermore, the overall architecture of the kidneys was less preserved in the absence of FtH [18]. These studies led to another interesting observation that FtH deletion was accompanied by markedly higher levels of heme oxygenase-1 (HO-1) expression. HO-1 is a well-characterized anti-oxidant enzyme that has been shown to have protective effects against a number insults and kidney injury models [73,74]. Aggravated AKI, despite such high levels of HO-1, indicates that beneficial effects of HO-1 are co-dependent on FtH expression and this premise is further supported in a model of rodent anti-thymocyte antigen-1-induced glomerulonephritis [75]. This study showed that inhibition of HO activity was accompanied with downregulation of FtH expression and enhanced mesangial cell death. Additionally, while induction of HO-1 in cultured rat mesangial cells augmented its resistance to oxidative stress, FtH knockdown by RNA interference caused loss of such protective influences. Also of note, this study demonstrated adjacent expression of HO-1 and FtH in inflammatory glomeruli of human lupus nephritis biopsies [75]. In another study, Hatcher and colleagues examined FtH overexpression in a model of ischemia-reperfusion-induced AKI [76]. Using a conditional tissue-specific doxycycline-inducible system, about 6.5-fold higher levels of FtH expression was shown in mouse kidneys. Following injury, the authors demonstrated that FtH overexpression was associated with lesser apoptosis and improved tubular viability [76]. It was concluded that FtH overexpression protects the kidney via limiting oxidative stress induced by I/R. Another line of evidence to support the role of FtH in protecting the kidney against injurious insults comes from a more recent study by Scindia and colleagues [77]. In this study, the authors examined how modulation of iron homeostatic pathways via hepcidin alters the course of I/R-induced AKI. Hepcidin, a hormone produced primarily by hepatocytes [78], acts by directly binding to ferroportin, leading to its internalization and subsequent degradation in lysosomes [79]. This eventually leads to decreased iron cation absorption, as well as increased iron cation retention with subsequent upregulation of intracellular ferritin. It was demonstrated that hepcidin, given 24–48 h before I/R, mitigated kidney injury and reduced renal and systemic inflammation caused by I/R [77]. These effects were attributed to increased FtH expression in the kidney and the spleen. These overall beneficial properties were also corroborated in a model of hemoglobin-induced AKI, where hepcidin administration mitigated the upregulation of urinary kidney injury markers (neutrophil gelatinase-associated lipocalin and kidney injury molecule-1) and renal Interleukin-6 [80]. 

To further elaborate on the role of FtH and how its expression in different cells may contribute to the course of kidney damage, effects of FtH expression in myeloid cells were further investigated [81]. Using transgenic mice with conditional deletion of FtH in the proximal tubules or myeloid cells, it was found that myeloid FtH deficiency did not affect activation or accumulation of macrophages in the injured kidney compared with wild-type littermate controls. In contrast, a significant increase in the number of pro-inflammatory macrophages accompanied FtH deletion in proximal tubules in a model of unilateral ureteral obstruction (UUO). Moreover, while deletion of FtH in the myeloid compartment resulted in lesser fibrosis in a UUO model of kidney injury, the lack of FtH expression in proximal tubules exacerbated both inflammation and fibrosis [81]. These findings suggest a central role for FtH expression in various compartments that underscores its importance in the context of tubular-macrophage cross-talk during kidney injury. More recently, the effects of FtH expression in myeloid cells during sepsis and its clinical sequelae, including sepsis-induced AKI, were examined [82]. Results of this study demonstrated that the deletion of FtH in the myeloid compartment was associated with marked protection in two models of sepsis, namely cecal ligation puncture and lipopolysaccharide-induced endotoxemia. Deletion of FtH led to improved survival, reduced cytokine levels, and more preserved renal function. Mechanistic studies revealed that the protective effects were primarily mediated by the compensatory increase in circulating ferritin (FtL) in the absence of myeloid FtH [82]. It must be noted that the main circulatory form of ferritin is FtL and macrophages have been shown to be the primary source of serum ferritin [11]. Results also demonstrated that the protective effects of FtL during sepsis are attributed to its inhibitory actions against the activation of the NF-κB pathway. These findings not only provide a novel platform for future studies to better understand the pathogenesis of sepsis but also shed light on the immunomodulatory roles of circulating FtL. 

## 3. Ferritin: A Potent Inhibitor of Osteoblastic Activity

Cardiovascular-related diseases remain the foremost cause of death in patients with CKD and those requiring renal replacement therapy [83,84,85]. About 10–15% of the U.S. population is estimated to have CKD and more than 450,000 Americans require dialysis resulting in a major burden of morbidity, mortality, and healthcare expenditure [86]. In this regard, vascular calcification (VC) is a common complication of CKD and is recognized as a portentous contributing factor to cardiovascular death in these patients [87,88,89,90]. The prevalence of VC is exceptionally high among patients with CKD and can be observed even in very young dialysis patients [91,92]. Two distinct patterns of VC have been described [93]. While intimal calcification occurs in atherosclerotic plaques, mineralization of the medial compartment is a common pathological finding in aging patients and patients with diabetes and advanced CKD [93,94]. The diffuse calcification that occurs in the medial layer of the vasculature is an important marker for all-cause mortality in patients undergoing dialysis [87,88,95]. There are several detrimental hemodynamic consequences of VC, including loss of arterial elasticity, increase in pulse wave velocity, development of left ventricular hypertrophy, and decrease in coronary artery perfusion, ensuing in myocardial ischemia and failure [95,96]. Following the description of VC as a tightly regulated cellular process where vascular smooth muscle cells (VSMC) transition into “osteoblast-like” cells, major research efforts have identified various pathways and mechanisms involved in the development and propagation of VC [97,98]. These proposed mechanisms include elastin degradation, apoptosis of VSMC, release of exosomes loaded with microRNAs, and extracellular vesicles that are rich in calcium and phosphate [99,100]. Nevertheless, multiple pivotal questions remain unanswered and currently no therapies exist for the treatment or prevention of VC. To better understand novel pathways that may be involved in the development and pathogenesis of VC, it was important to examine how derangements in iron metabolism may be contributing to the development of such detrimental pathological conditions. This principle was based on the fact that many patients with advanced CKD are also predisposed to iron-restricted erythropoiesis. Several factors lead to the development of anemia in patients with CKD and those requiring dialysis. These include decreased erythropoietin production, frequent diagnostic testing, blood loss during hemodialysis, and cannula puncture sites following hemodialysis, as well as a “functional iron deficiency state” [101,102,103,104]. This process, also known as anemia of chronic disease, is principally a result of the increased synthesis and secretion of hepcidin, which is a common paradigm of chronic inflammatory states such as CKD [105]. It must be noted that other cytokines have been demonstrated to have additional effects and further aggravate this state of functional iron deficiency [106]. Hepcidin leads to the internalization and degradation of ferroportin (membrane iron transporter) and the overall outcome is the decreased iron cation absorption from the small intestine along with the decreased egress of iron cations from the reticuloendothelial system [105]. Accordingly, while macrophages are loaded with iron cations, other cells within the body have a relatively lower iron cation content. It was therefore necessary to examine whether the repletion of iron cations and the subsequent upregulation of ferritin in VSMC mitigates the osteoblastic differentiation of these cells when exposed to high phosphate levels. Notably, increasing levels of serum phosphorus manifest during advanced stages of CKD and such hyperphosphatemia is a well-known risk factor for the development of VC [107,108,109]. Indeed, we found that the addition of hemin was able to significantly arrest the calcification and osteoblastic transition of VSMC [110]. 

To identify the mediator of such a salutary effect, we examined products of the HO/ferritin system that include biliverdin, bilirubin, carbon monoxide, and iron cations. It was demonstrated that iron administration inhibited calcium deposition and led to about a five-fold increment in intracellular ferritin levels. Subsequent studies using recombinant ferritin proteins confirmed the paramount role of ferritin upregulation in this inhibitory process. Using exogenous recombinant FtH, FtL, and a mutant form of FtH that lacks ferroxidase activity, we demonstrated the paramount role of FtH/ferroxidase activity in the abrogation of VSMC mineralization and osteoblastic transformation [110]. Importantly, the inhibitory role of FtH was not limited to the mere inhibition of hydroxyapatite deposition, but it also prevented the expression of the core binding factor alpha-1 (cbfa-1), a key transcription factor in osteogenesis [111]. 

Other studies have also suggested a potential inhibitory role for iron and iron-induced upregulation of ferritin in the context of VC. For example, Rajendran et al. used various detection methods to map the spatial distribution of the elements and quantify them simultaneously in atherosclerotic rabbit arteries [112]. They demonstrated that within atherosclerotic plaques, iron cations (likely sequestered within ferritin shell) and calcium exhibited a highly significant spatial inverse correlation. Moreover, an adenine-induced model of CKD in rats was employed to investigate the role of PA-21, an iron-based non-calcium phosphate binder in VC. They reported a higher degree of inhibition of VC when compared to another frequently used phosphate binder, calcium carbonate [113]. Furthermore, Seto et al. examined the role of iron loading on the progression of VC in an adenine diet-induced CKD rat model [114]. It was demonstrated that iron loading resulted in the suppression of VC and attributed these findings to the reduced expression of phosphate transporter (Pit-1) and cbfa-1. Others have also reported the inhibition of high phosphate-induced VC using ferric citrate and attributed these results to the suppression of apoptosis [115]. These results are interesting when taking into account a study that was published in 1995 and reported the inhibition of calcium deposition in bioprostethic valves when pre-treated with iron [116]. However, it is also noteworthy that some studies have failed to recapitulate the overall inhibitory effects of iron during VC [117]. 

Based on these findings, we hypothesized that osteoporosis induced by iron overload states, such as primary hemochromatosis [118,119], may be attributed to the increased expression of FtH in osteoblasts secondary to excessive iron accumulation. Indeed, investigating the mechanism(s) leading to decreased bone deposition in iron overload states, we were able to demonstrate the central role of FtH in this process [120]. We showed that the administration of apoferritin or recombinant FtH (both devoid of iron cations) markedly inhibited the mineralization and expression of osteoblastic specific genes, such as osteocalcin, alkaline phosphatase, and cbfa-1 [120]. Overall these results confirmed previous findings that increased iron cation levels mitigate calcification, but they also provided a novel mechanism for this process and identified FtH upregulation as a main inhibitory factor in this regard. 

**Figure 1:**
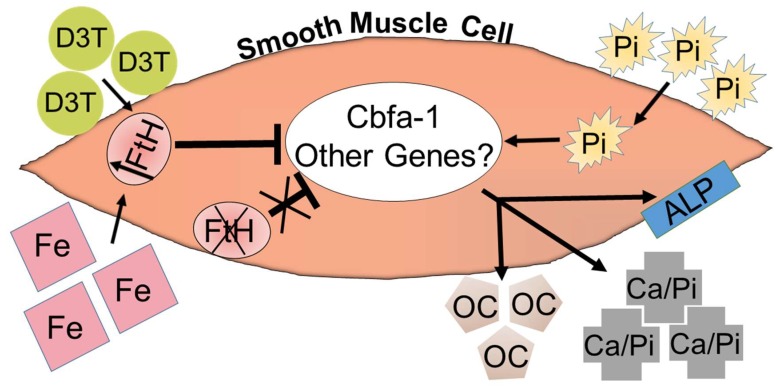
Overall schematic of the proposed mechanism of the inhibition of VSMC calcification via the upregulation of FtH. The figure depicts a vascular smooth muscle cell (VSMC) where elevated levels of Pi (phosphorus) induce the upregulation of cbfa-1 (core binding factor alpha-1), leading to the deposition of extracellular Ca/Pi (hydroxyapatite crystals) and the activation of osteoblastic genes, including OC (osteocalcin) and ALP (alkaline phosphatase). This process can be exacerbated by the deletion of FtH (ferritin heavy chain) or mitigated via D3T (3H-1,2-Dithiole-3-thione) and Fe (iron)-induced FtH upregulation. There may still be novel genes and pathways modulated by FtH expression that require further investigations.

The potential hazardous effects of excessive iron cations are well recognized, and their optimal dosage and utilization frequency in CKD patients have been a major point of debate among nephrologists [121,122,123]. Accordingly, based on the aforementioned evidence, we sought to identify other approaches to upregulate FtH expression in VSMC without using excessive iron and examine how the pharmacological induction of ferritin may affect VC. To achieve this objective, we tested the effects of 3H-1,2-Dithiole-3-thione (D3T), a well-known cancer chemopreventive agent [124] and inducer of FtH expression [125], in a model of VSMC calcification. It was demonstrated that D3T inhibited osteoblastic transition of VSMC in a dose-dependent manner [126]. However, the central role of ferritin was further corroborated in experiments where the inhibitory effects of D3T on osteoblastic transition were arrested during FtH knockdown by RNA interference [126]. The overall effect of iron/D3T-induced FtH upregulation and inhibition of VSMC calcification is illustrated in Figure 1. 

More recently, similar inhibitory effects in mitigating the calcification of valvular tissues were demonstrated [127]. Valvular calcification and stenosis (particularly aortic valve) is a common finding in patients undergoing dialysis and the elderly population. In this study we showed that the induction of FtH was associated with the decreased osteoblastic activity of valvular interstitial cells. This inhibitory effect was attributed to the reduced nuclear accumulation of cbfa-1, and as a reciprocal effect, its enhancement of the nuclear localization of transcription factor Sox9 (SRY [sex-determining region Y]-box 9) [127]. These findings provide additional support to previous observations that identified reduced Sox9 function as a potential culprit of calcific valvular disease [128] and further corroborate the “anti-osteoblastic activity” of FtH. It must be emphasized that while a significant body of evidence is emerging to support the role of intracellular FtH expression as an inhibitory mechanism against calcification, additional targeted studies in animal models are required to unequivocally demonstrate and confirm these results. 

It is also noteworthy that the implication and functional significance of iron and ferritin in the context of atherosclerosis and coronary artery disease continues to generate contradicting results and is heavily debated in the literature. While we do not elaborate further on these studies in this review, others have discussed these results in detail [129,130,131,132,133,134]

In conclusion, it is evident that ferritin and its role in both cellular and systemic iron homeostasis, as well as its participation in a number of pathways that are central to various pathological conditions, is increasingly recognized. Future studies and additional genetic manipulations (for instance, the targeted deletion of FtL or the over-expression of FtH) would be germane to pave the way for potential therapeutic targets utilizing this intriguing, ancient, and almost ubiquitous superfamily of proteins.

## References

[1] Haber F., Weiss J. (1934). The Catalytic Decomposition of Hydrogen Peroxide by Iron Salts. Proc. R. Soc. Lond. Ser. A Math. Phys. Sci..

[2] Fenton H.J.H. (1894). LXXIII.—Oxidation of tartaric acid in presence of iron. J. Chem. Soc. Trans..

[3] Laufberger V. (1937). Sur la cristallisation de la ferritine. Bull. Soc. Chim. Biol..

[4] Arosio P., Ingrassia R., Cavadini P. (2009). Ferritins: A family of molecules for iron storage, antioxidation and more. Biochim. Biophys. Acta.

[5] Arosio P., Levi S. (2010). Cytosolic and mitochondrial ferritins in the regulation of cellular iron homeostasis and oxidative damage. Biochim. Biophys. Acta.

[6] Arosio P., Levi S. (2002). Ferritin, iron homeostasis, and oxidative damage. Free Radic. Biol. Med..

[7] Harrison P.M., Arosio P. (1996). The ferritins: Molecular properties, iron storage function and cellular regulation. Biochim. Biophys. Acta.

[8] Santambrogio P., Cozzi A., Levi S., Arosio P. (1987). Human serum ferritin G-peptide is recognized by anti-L ferritin subunit antibodies and concanavalin-A. Br. J. Haematol..

[9] Cazzola M., Arosio P., Bellotti V., Bergamaschi G., Dezza L., Iacobello C., Ruggeri G., Zappone E., Albertini A., Ascari E. (1985). Immunological reactivity of serum ferritin in patients with malignancy. Tumori.

[10] Lukina E.A., Levina A.A., Mokeeva R.A., Tokarev Yu N. (1993). The diagnostic significance of serum ferritin indices in patients with malignant and reactive histiocytosis. Br. J. Haematol..

[11] Cohen L.A., Gutierrez L., Weiss A., Leichtmann-Bardoogo Y., Zhang D.L., Crooks D.R., Sougrat R., Morgenstern A., Galy B., Hentze M.W. (2010). Serum ferritin is derived primarily from macrophages through a nonclassical secretory pathway. Blood.

[12] Ghosh S., Hevi S., Chuck S.L. (2004). Regulated secretion of glycosylated human ferritin from hepatocytes. Blood.

[13] Mack U., Cooksley W.G., Ferris R.A., Powell L.W., Halliday J.W. (1981). Regulation of plasma ferritin by the isolated perfused rat liver. Br. J. Haematol..

[14] Tran T.N., Eubanks S.K., Schaffer K.J., Zhou C.Y., Linder M.C. (1997). Secretion of ferritin by rat hepatoma cells and its regulation by inflammatory cytokines and iron. Blood.

[15] Truman-Rosentsvit M., Berenbaum D., Spektor L., Cohen L.A., Belizowsky-Moshe S., Lifshitz L., Ma J., Li W., Kesselman E., Abutbul-Ionita I. (2018). Ferritin is secreted via 2 distinct nonclassical vesicular pathways. Blood.

[16] Kalantar-Zadeh K., Kalantar-Zadeh K., Lee G.H. (2006). The fascinating but deceptive ferritin: To measure it or not to measure it in chronic kidney disease?. Clin. J. Am. Soc. Nephrol..

[17] Zandman-Goddard G., Shoenfeld Y. (2007). Ferritin in autoimmune diseases. Autoimmun. Rev..

[18] Zarjou A., Bolisetty S., Joseph R., Traylor A., Apostolov E.O., Arosio P., Balla J., Verlander J., Darshan D., Kuhn L.C. (2013). Proximal tubule H-ferritin mediates iron trafficking in acute kidney injury. J. Clin. Invest..

[19] Wang W., Knovich M.A., Coffman L.G., Torti F.M., Torti S.V. (2010). Serum ferritin: Past, present and future. Biochim. Biophys. Acta.

[20] Bahr T.M., Christensen R.D., Ward D.M., Meng F., Jackson L.K., Doyle K., Christensen D.R., Harvey A.G., Yaish H.M. (2019). Ferritin in serum and urine: A pilot study. Blood Cells Mol. Dis..

[21] Ishikawa K., Narita O., Saito H., Kato K. (1982). Determination of ferritin in urine and in serum of normal adults with a sensitive enzyme immunoassay. Clin. Chim. Acta.

[22] Gonzales P.A., Pisitkun T., Hoffert J.D., Tchapyjnikov D., Star R.A., Kleta R., Wang N.S., Knepper M.A. (2009). Large-scale proteomics and phosphoproteomics of urinary exosomes. J. Am. Soc. Nephrol..

[23] Hentze M.W., Keim S., Papadopoulos P., O’Brien S., Modi W., Drysdale J., Leonard W.J., Harford J.B., Klausner R.D. (1986). Cloning, characterization, expression, and chromosomal localization of a human ferritin heavy-chain gene. Proc. Natl. Acad. Sci. USA.

[24] Lawson D.M., Treffry A., Artymiuk P.J., Harrison P.M., Yewdall S.J., Luzzago A., Cesareni G., Levi S., Arosio P. (1989). Identification of the ferroxidase centre in ferritin. FEBS Lett..

[25] Arosio P., Elia L., Poli M. (2017). Ferritin, cellular iron storage and regulation. IUBMB Life.

[26] Watt R.K. (2011). The many faces of the octahedral ferritin protein. Biometals.

[27] Balla G., Jacob H.S., Balla J., Rosenberg M., Nath K., Apple F., Eaton J.W., Vercellotti G.M. (1992). Ferritin: A cytoprotective antioxidant strategem of endothelium. J. Biol. Chem..

[28] Levi S., Corsi B., Bosisio M., Invernizzi R., Volz A., Sanford D., Arosio P., Drysdale J. (2001). A human mitochondrial ferritin encoded by an intronless gene. J. Biol. Chem..

[29] Drysdale J., Arosio P., Invernizzi R., Cazzola M., Volz A., Corsi B., Biasiotto G., Levi S. (2002). Mitochondrial ferritin: A new player in iron metabolism. Blood Cells Mol. Dis..

[30] Cai C.X., Birk D.E., Linsenmayer T.F. (1998). Nuclear ferritin protects DNA from UV damage in corneal epithelial cells. Mol. Biol. Cell.

[31] Cai C., Ching A., Lagace C., Linsenmayer T. (2008). Nuclear ferritin-mediated protection of corneal epithelial cells from oxidative damage to DNA. Dev. Dyn..

[32] Thompson K.J., Fried M.G., Ye Z., Boyer P., Connor J.R. (2002). Regulation, mechanisms and proposed function of ferritin translocation to cell nuclei. J. Cell Sci..

[33] Zarjou A., Sanders P.W., Mehta R.L., Agarwal A. (2012). Enabling innovative translational research in acute kidney injury. Clin. Transl. Sci..

[34] Choudhury D. (2010). Acute kidney injury: Current perspectives. Postgrad. Med..

[35] Maxwell R.A., Bell C.M. (2017). Acute Kidney Injury in the Critically Ill. Surg. Clin. N. Am..

[36] Bevc S., Ekart R., Hojs R. (2017). The assessment of acute kidney injury in critically ill patients. Eur. J. Intern. Med..

[37] Chawla L.S., Eggers P.W., Star R.A., Kimmel P.L. (2014). Acute kidney injury and chronic kidney disease as interconnected syndromes. N. Engl. J. Med..

[38] Basile D.P., Donohoe D., Roethe K., Osborn J.L. (2001). Renal ischemic injury results in permanent damage to peritubular capillaries and influences long-term function. Am. J. Physiol. Ren. Physiol..

[39] Chawla L.S., Kimmel P.L. (2012). Acute kidney injury and chronic kidney disease: An integrated clinical syndrome. Kidney Int..

[40] Cutillas P.R., Chalkley R.J., Hansen K.C., Cramer R., Norden A.G., Waterfield M.D., Burlingame A.L., Unwin R.J. (2004). The urinary proteome in Fanconi syndrome implies specificity in the reabsorption of proteins by renal proximal tubule cells. Am. J. Physiol. Ren. Physiol..

[41] Norden A.G., Lapsley M., Lee P.J., Pusey C.D., Scheinman S.J., Tam F.W., Thakker R.V., Unwin R.J., Wrong O. (2001). Glomerular protein sieving and implications for renal failure in Fanconi syndrome. Kidney Int..

[42] Kozyraki R., Fyfe J., Verroust P.J., Jacobsen C., Dautry-Varsat A., Gburek J., Willnow T.E., Christensen E.I., Moestrup S.K. (2001). Megalin-dependent cubilin-mediated endocytosis is a major pathway for the apical uptake of transferrin in polarized epithelia. Proc. Natl. Acad. Sci. USA.

[43] Smith C.P., Lee W.K., Haley M., Poulsen S.B., Thevenod F., Fenton R.A. (2019). Proximal tubule transferrin uptake is modulated by cellular iron and mediated by apical membrane megalin-cubilin complex and transferrin receptor 1. J. Biol. Chem..

[44] Gunshin H., Mackenzie B., Berger U.V., Gunshin Y., Romero M.F., Boron W.F., Nussberger S., Gollan J.L., Hediger M.A. (1997). Cloning and characterization of a mammalian proton-coupled metal-ion transporter. Nature.

[45] Tchernitchko D., Bourgeois M., Martin M.E., Beaumont C. (2002). Expression of the two mRNA isoforms of the iron transporter Nramp2/DMTI in mice and function of the iron responsive element. Biochem. J..

[46] Abouhamed M., Gburek J., Liu W., Torchalski B., Wilhelm A., Wolff N.A., Christensen E.I., Thevenod F., Smith C.P. (2006). Divalent metal transporter 1 in the kidney proximal tubule is expressed in late endosomes/lysosomal membranes: Implications for renal handling of protein-metal complexes. Am. J. Physiol. Ren. Physiol..

[47] Wareing M., Ferguson C.J., Delannoy M., Cox A.G., McMahon R.F., Green R., Riccardi D., Smith C.P. (2003). Altered dietary iron intake is a strong modulator of renal DMT1 expression. Am. J. Physiol. Ren. Physiol..

[48] Ferguson C.J., Wareing M., Ward D.T., Green R., Smith C.P., Riccardi D. (2001). Cellular localization of divalent metal transporter DMT-1 in rat kidney. Am. J. Physiol. Ren. Physiol..

[49] Ferguson C.J., Wareing M., Delannoy M., Fenton R., McLarnon S.J., Ashton N., Cox A.G., McMahon R.F., Garrick L.M., Green R. (2003). Iron handling and gene expression of the divalent metal transporter, DMT1, in the kidney of the anemic Belgrade (b) rat. Kidney Int..

[50] Mori K., Lee H.T., Rapoport D., Drexler I.R., Foster K., Yang J., Schmidt-Ott K.M., Chen X., Li J.Y., Weiss S. (2005). Endocytic delivery of lipocalin-siderophore-iron complex rescues the kidney from ischemia-reperfusion injury. J. Clin. Investig..

[51] Donovan A., Brownlie A., Zhou Y., Shepard J., Pratt S.J., Moynihan J., Paw B.H., Drejer A., Barut B., Zapata A. (2000). Positional cloning of zebrafish ferroportin1 identifies a conserved vertebrate iron exporter. Nature.

[52] Wolff N.A., Liu W., Fenton R.A., Lee W.K., Thevenod F., Smith C.P. (2011). Ferroportin 1 is expressed basolaterally in rat kidney proximal tubule cells and iron excess increases its membrane trafficking. J. Cell Mol. Med..

[53] Kulaksiz H., Theilig F., Bachmann S., Gehrke S.G., Rost D., Janetzko A., Cetin Y., Stremmel W. (2005). The iron-regulatory peptide hormone hepcidin: Expression and cellular localization in the mammalian kidney. J. Endocrinol..

[54] Meyron-Holtz E.G., Ghosh M.C., Iwai K., LaVaute T., Brazzolotto X., Berger U.V., Land W., Ollivierre-Wilson H., Grinberg A., Love P. (2004). Genetic ablations of iron regulatory proteins 1 and 2 reveal why iron regulatory protein 2 dominates iron homeostasis. EMBO J..

[55] Smith C.P., Thevenod F. (2009). Iron transport and the kidney. Biochim. Biophys. Acta.

[56] Leaf D.E., Swinkels D.W. (2016). Catalytic iron and acute kidney injury. Am. J. Physiol. Ren. Physiol..

[57] Walker V.J., Agarwal A. (2016). Targeting Iron Homeostasis in Acute Kidney Injury. Semin. Nephrol..

[58] Martines A.M., Masereeuw R., Tjalsma H., Hoenderop J.G., Wetzels J.F., Swinkels D.W. (2013). Iron metabolism in the pathogenesis of iron-induced kidney injury. Nat. Rev. Nephrol..

[59] Swaminathan S. (2018). Iron Homeostasis Pathways as Therapeutic Targets in Acute Kidney Injury. Nephron.

[60] Shah S.V., Walker P.D. (1988). Evidence suggesting a role for hydroxyl radical in glycerol-induced acute renal failure. Am. J. Physiol..

[61] Paller M.S. (1988). Hemoglobin- and myoglobin-induced acute renal failure in rats: Role of iron in nephrotoxicity. Am. J. Physiol..

[62] Walker P.D., Shah S.V. (1988). Evidence suggesting a role for hydroxyl radical in gentamicin-induced acute renal failure in rats. J. Clin. Investig..

[63] Paller M.S., Hedlund B.E. (1988). Role of iron in postischemic renal injury in the rat. Kidney Int..

[64] De Vries B., Walter S.J., von Bonsdorff L., Wolfs T.G., van Heurn L.W., Parkkinen J., Buurman W.A. (2004). Reduction of circulating redox-active iron by apotransferrin protects against renal ischemia-reperfusion injury. Transplantation.

[65] Leaf D.E., Rajapurkar M., Lele S.S., Mukhopadhyay B., Rawn J.D., Frendl G., Waikar S.S. (2015). Increased plasma catalytic iron in patients may mediate acute kidney injury and death following cardiac surgery. Kidney Int..

[66] Leaf D.E., Rajapurkar M., Lele S.S., Mukhopadhyay B., Waikar S.S. (2014). Plasma catalytic iron, AKI, and death among critically ill patients. Clin. J. Am. Soc. Nephrol..

[67] Lele S.S., Mukhopadhyay B.N., Mardikar M.M., Patel T.A., Vasavada A.K., Banker D.N., Kapasi K.D., Chauhan V.C., Chawla K.C., Raju S.R. (2013). Impact of catalytic iron on mortality in patients with acute coronary syndrome exposed to iodinated radiocontrast-The Iscom Study. Am. Heart J..

[68] Ueda N., Baliga R., Shah S.V. (1996). Role of ‘catalytic’ iron in an animal model of minimal change nephrotic syndrome. Kidney Int..

[69] Rajapurkar M.M., Hegde U., Bhattacharya A., Alam M.G., Shah S.V. (2013). Effect of deferiprone, an oral iron chelator, in diabetic and non-diabetic glomerular disease. Toxicol. Mech. Methods.

[70] Ferreira C., Bucchini D., Martin M.E., Levi S., Arosio P., Grandchamp B., Beaumont C. (2000). Early embryonic lethality of H ferritin gene deletion in mice. J. Biol. Chem..

[71] Darshan D., Vanoaica L., Richman L., Beermann F., Kuhn L.C. (2009). Conditional deletion of ferritin H in mice induces loss of iron storage and liver damage. Hepatology.

[72] Rankin E.B., Tomaszewski J.E., Haase V.H. (2006). Renal cyst development in mice with conditional inactivation of the von Hippel-Lindau tumor suppressor. Cancer Res..

[73] Bolisetty S., Zarjou A., Agarwal A. (2017). Heme Oxygenase 1 as a Therapeutic Target in Acute Kidney Injury. Am. J. Kidney Dis..

[74] Ayer A., Zarjou A., Agarwal A., Stocker R. (2016). Heme Oxygenases in Cardiovascular Health and Disease. Physiol. Rev..

[75] Cheng H.T., Yen C.J., Chang C.C., Huang K.T., Chen K.H., Zhang R.Y., Lee P.Y., Miaw S.C., Huang J.W., Chiang C.K. (2015). Ferritin heavy chain mediates the protective effect of heme oxygenase-1 against oxidative stress. Biochim. Biophys. Acta.

[76] Hatcher H.C., Tesfay L., Torti S.V., Torti F.M. (2015). Cytoprotective Effect of Ferritin H in Renal Ischemia Reperfusion Injury. PLoS ONE.

[77] Scindia Y., Dey P., Thirunagari A., Liping H., Rosin D.L., Floris M., Okusa M.D., Swaminathan S. (2015). Hepcidin Mitigates Renal Ischemia-Reperfusion Injury by Modulating Systemic Iron Homeostasis. J. Am. Soc. Nephrol..

[78] Roetto A., Papanikolaou G., Politou M., Alberti F., Girelli D., Christakis J., Loukopoulos D., Camaschella C. (2003). Mutant antimicrobial peptide hepcidin is associated with severe juvenile hemochromatosis. Nat. Genet..

[79] Girelli D., Nemeth E., Swinkels D.W. (2016). Hepcidin in the diagnosis of iron disorders. Blood.

[80] Van Swelm R.P., Wetzels J.F., Verweij V.G., Laarakkers C.M., Pertijs J.C., van der Wijst J., Thevenod F., Masereeuw R., Swinkels D.W. (2016). Renal Handling of Circulating and Renal-Synthesized Hepcidin and Its Protective Effects against Hemoglobin-Mediated Kidney Injury. J. Am. Soc. Nephrol..

[81] Bolisetty S., Zarjou A., Hull T.D., Traylor A.M., Perianayagam A., Joseph R., Kamal A.I., Arosio P., Soares M.P., Jeney V. (2015). Macrophage and epithelial cell H-ferritin expression regulates renal inflammation. Kidney Int..

[82] Zarjou A., Black L.M., McCullough K.R., Hull T.D., Esman S.K., Boddu R., Varambally S., Chandrashekar D.S., Feng W., Arosio P. (2019). Ferritin Light Chain Confers Protection Against Sepsis-Induced Inflammation and Organ Injury. Front. Immunol..

[83] Schiffrin E.L., Lipman M.L., Mann J.F. (2007). Chronic kidney disease: Effects on the cardiovascular system. Circulation.

[84] Bloembergen W.E. (1997). Cardiac disease in chronic uremia: Epidemiology. Adv. Ren. Replace. Ther..

[85] Herzog C.A., Asinger R.W., Berger A.K., Charytan D.M., Diez J., Hart R.G., Eckardt K.U., Kasiske B.L., McCullough P.A., Passman R.S. (2011). Cardiovascular disease in chronic kidney disease. A clinical update from Kidney Disease: Improving Global Outcomes (KDIGO). Kidney Int..

[86] Norton J.M., Newman E.P., Romancito G., Mahooty S., Kuracina T., Narva A.S. (2017). CE: Improving Outcomes for Patients with Chronic Kidney Disease: Part 1. Am. J. Nurs..

[87] Covic A., Kanbay M., Voroneanu L., Turgut F., Serban D.N., Serban I.L., Goldsmith D.J. (2010). Vascular calcification in chronic kidney disease. Clin. Sci. (Lond.).

[88] McIntyre C.W. (2007). The functional cardiovascular consequences of vascular calcification. Semin. Dial..

[89] London G.M., Marchais S.J., Guerin A.P., Metivier F. (2005). Arteriosclerosis, vascular calcifications and cardiovascular disease in uremia. Curr. Opin. Nephrol. Hypertens..

[90] Gusbeth-Tatomir P., Covic A. (2007). Causes and consequences of increased arterial stiffness in chronic kidney disease patients. Kidney Blood Press Res..

[91] Goodman W.G., Goldin J., Kuizon B.D., Yoon C., Gales B., Sider D., Wang Y., Chung J., Emerick A., Greaser L. (2000). Coronary-artery calcification in young adults with end-stage renal disease who are undergoing dialysis. N. Engl. J. Med..

[92] Lumpaopong A., Mathew A.V., John E., Jelnin V., Benedetti E., Testa G., Oberholzer J., Sankary H., Ruiz C. (2007). Early coronary calcification in children and young adults with end-stage renal disease. Transplant. Proc..

[93] Jono S., Shioi A., Ikari Y., Nishizawa Y. (2006). Vascular calcification in chronic kidney disease. J. Bone Miner. Metab..

[94] Yamada S., Giachelli C.M. (2017). Vascular calcification in CKD-MBD: Roles for phosphate, FGF23, and Klotho. Bone.

[95] Mizobuchi M., Towler D., Slatopolsky E. (2009). Vascular calcification: The killer of patients with chronic kidney disease. J. Am. Soc. Nephrol..

[96] Zhu D., Mackenzie N.C., Farquharson C., Macrae V.E. (2012). Mechanisms and clinical consequences of vascular calcification. Front. Endocrinol. (Lausanne).

[97] El-Abbadi M., Giachelli C.M. (2007). Mechanisms of vascular calcification. Adv. Chronic Kidney Dis..

[98] Shroff R.C., Shanahan C.M. (2007). The vascular biology of calcification. Semin. Dial..

[99] McCarty M.F., DiNicolantonio J.J. (2014). The molecular biology and pathophysiology of vascular calcification. Postgrad. Med..

[100] Liberman M., Marti L.C. (2017). Vascular Calcification Regulation by Exosomes in the Vascular Wall. Adv. Exp. Med. Biol..

[101] Zarychanski R., Houston D.S. (2008). Anemia of chronic disease: A harmful disorder or an adaptive, beneficial response?. CMAJ.

[102] Webster A.C., Nagler E.V., Morton R.L., Masson P. (2017). Chronic Kidney Disease. Lancet.

[103] Panwar B., Gutierrez O.M. (2016). Disorders of Iron Metabolism and Anemia in Chronic Kidney Disease. Semin. Nephrol..

[104] Babitt J.L., Lin H.Y. (2012). Mechanisms of anemia in CKD. J. Am. Soc. Nephrol..

[105] Ueda N., Takasawa K. (2017). Role of Hepcidin-25 in Chronic Kidney Disease: Anemia and Beyond. Curr. Med. Chem..

[106] Cassat J.E., Skaar E.P. (2013). Iron in infection and immunity. Cell Host Microbe.

[107] Fuery M.A., Liang L., Kaplan F.S., Mohler E.R. (2018). Vascular ossification: Pathology, mechanisms, and clinical implications. Bone.

[108] Burke S.K. (2008). Phosphate is a uremic toxin. J. Ren. Nutr..

[109] Block G.A., Klassen P.S., Lazarus J.M., Ofsthun N., Lowrie E.G., Chertow G.M. (2004). Mineral metabolism, mortality, and morbidity in maintenance hemodialysis. J. Am. Soc. Nephrol..

[110] Zarjou A., Jeney V., Arosio P., Poli M., Antal-Szalmas P., Agarwal A., Balla G., Balla J. (2009). Ferritin prevents calcification and osteoblastic differentiation of vascular smooth muscle cells. J. Am. Soc. Nephrol..

[111] Lian J.B., Stein G.S. (2003). Runx2/Cbfa1: A multifunctional regulator of bone formation. Curr. Pharm. Des..

[112] Rajendran R., Minqin R., Ronald J.A., Rutt B.K., Halliwell B., Watt F. (2012). Does iron inhibit calcification during atherosclerosis?. Free Radic. Biol. Med..

[113] Phan O., Maillard M., Peregaux C., Mordasini D., Stehle J.C., Funk F., Burnier M. (2013). PA21, a new iron-based noncalcium phosphate binder, prevents vascular calcification in chronic renal failure rats. J. Pharmacol. Exp. Ther..

[114] Seto T., Hamada C., Tomino Y. (2014). Suppressive effects of iron overloading on vascular calcification in uremic rats. J. Nephrol..

[115] Ciceri P., Elli F., Braidotti P., Falleni M., Tosi D., Bulfamante G., Block G.A., Cozzolino M. (2016). Iron citrate reduces high phosphate-induced vascular calcification by inhibiting apoptosis. Atherosclerosis.

[116] Carpentier S.M., Carpentier A.F., Chen L., Shen M., Quintero L.J., Witzel T.H. (1995). Calcium mitigation in bioprosthetic tissues by iron pretreatment: The challenge of iron leaching. Ann. Thorac. Surg..

[117] Neven E., De Schutter T.M., Behets G.J., Gupta A., D’Haese P.C. (2011). Iron and vascular calcification. Is there a link?. Nephrol. Dial. Transplant..

[118] Balogh E., Paragh G., Jeney V. (2018). Influence of Iron on Bone Homeostasis. Pharmaceuticals (Basel).

[119] Jeney V. (2017). Clinical Impact and Cellular Mechanisms of Iron Overload-Associated Bone Loss. Front. Pharmacol..

[120] Zarjou A., Jeney V., Arosio P., Poli M., Zavaczki E., Balla G., Balla J. (2010). Ferritin ferroxidase activity: A potent inhibitor of osteogenesis. J. Bone Miner. Res..

[121] Fishbane S., Mathew A., Vaziri N.D. (2014). Iron toxicity: Relevance for dialysis patients. Nephrol. Dial. Transplant..

[122] Afzali B., Goldsmith D.J. (2004). Intravenous iron therapy in renal failure: Friend and foe?. J. Nephrol..

[123] Brewster U.C. (2006). Intravenous iron therapy in end-stage renal disease. Semin. Dial..

[124] Zhang Y., Munday R. (2008). Dithiolethiones for cancer chemoprevention: Where do we stand?. Mol. Cancer Ther..

[125] Pietsch E.C., Chan J.Y., Torti F.M., Torti S.V. (2003). Nrf2 mediates the induction of ferritin H in response to xenobiotics and cancer chemopreventive dithiolethiones. J. Biol. Chem..

[126] Becs G., Zarjou A., Agarwal A., Kovacs K.E., Becs A., Nyitrai M., Balogh E., Banyai E., Eaton J.W., Arosio P. (2016). Pharmacological induction of ferritin prevents osteoblastic transformation of smooth muscle cells. J. Cell. Mol. Med..

[127] Sikura K.E., Potor L., Szerafin T., Zarjou A., Agarwal A., Arosio P., Poli M., Hendrik Z., Mehes G., Oros M. (2019). Potential Role of H-Ferritin in Mitigating Valvular Mineralization. Arterioscler. Thromb. Vasc. Biol..

[128] Peacock J.D., Levay A.K., Gillaspie D.B., Tao G., Lincoln J. (2010). Reduced sox9 function promotes heart valve calcification phenotypes in vivo. Circ. Res..

[129] Kraml P. (2017). The role of iron in the pathogenesis of atherosclerosis. Physiol. Res..

[130] Das De S., Krishna S., Jethwa A. (2015). Iron status and its association with coronary heart disease: Systematic review and meta-analysis of prospective studies. Atherosclerosis.

[131] Silvestre O.M., Goncalves A., Nadruz W., Claggett B., Couper D., Eckfeldt J.H., Pankow J.S., Anker S.D., Solomon S.D. (2017). Ferritin levels and risk of heart failure-the Atherosclerosis Risk in Communities Study. Eur. J. Heart Fail..

[132] Galesloot T.E., Janss L.L., Burgess S., Kiemeney L.A., den Heijer M., de Graaf J., Holewijn S., Benyamin B., Whitfield J.B., Swinkels D.W. (2015). Iron and hepcidin as risk factors in atherosclerosis: What do the genes say?. BMC Genet..

[133] Lapice E., Masulli M., Vaccaro O. (2013). Iron deficiency and cardiovascular disease: An updated review of the evidence. Curr. Atheroscler. Rep..

[134] Sullivan J.L. (2009). Do hemochromatosis mutations protect against iron-mediated atherogenesis?. Circ. Cardiovasc. Genet..

